# Association between early-pregnancy serum C-peptide and risk of gestational diabetes mellitus: a nested case–control study among Chinese women

**DOI:** 10.1186/s12986-022-00691-3

**Published:** 2022-08-22

**Authors:** Xue Yang, Yi Ye, Yi Wang, Ping Wu, Qi Lu, Yan Liu, Jiaying Yuan, Xingyue Song, Shijiao Yan, Xiaorong Qi, Yi-Xin Wang, Ying Wen, Gang Liu, Chuanzhu Lv, Chun-Xia Yang, An Pan, Jianli Zhang, Xiong-Fei Pan

**Affiliations:** 1 Department of Epidemiology and Biostatistics, West China School of Public Health and West China Fourth Hospital, Sichuan University, Chengdu, 610041 Sichuan China; 2 Non-Communicable Diseases Research Center, West China-PUMC C.C. Chen Institute of Health, Sichuan University, Chengdu, 610041 Sichuan China; 3 Wenjiang Institute of Women’s and Children’s Health, Wenjiang Maternal and Child Health Hospital, Chengdu, 611130 Sichuan China; 4 Department of Epidemiology and Biostatistics, Tongji Medical College, Huazhong University of Science and Technology, Wuhan, 430030 Hubei China; 5 Ministry of Education and Ministry of Environmental Protection Key Laboratory of Environment and Health, and State Key Laboratory of Environmental Health (Incubation), School of Public Health, Tongji Medical College, Huazhong University of Science and Technology, Wuhan, 430030 Hubei China; 6 Department of Obstetrics and Gynecology, Shuangliu Maternal and Child Health Hospital, Chengdu, 610200 Sichuan China; 7 Department of Science and Education, Shuangliu Maternal and Child Health Hospital, Chengdu, 610200 Sichuan China; 8 Department of Emergency, Hainan Clinical Research Center for Acute and Critical Diseases, The Second Affiliated Hospital of Hainan Medical University, Haikou, 571199 Hainan China; 9 Key Laboratory of Emergency and Trauma of Ministry of Education, Hainan Medical University, Haikou, 571199 Hainan China; 10 Research Unit of Island Emergency Medicine, Chinese Academy of Medical Sciences, Hainan Medical University, Haikou, 571199 Hainan China; 11 School of Public Health, Hainan Medical University, Haikou, 571199 Hainan China; 12 Department of Gynecology and Obstetrics, West China Second Hospital, State Key Laboratory of Biotherapy, Sichuan University, Chengdu, 610041 Sichuan China; 13 Department of Nutrition, Harvard TH Chan School of Public Health, Boston, MA 02115 USA; 14 Department of Communicable Diseases Control and Prevention, Shenzhen Center for Disease Control and Prevention, Shenzhen, 518055 Guangdong China; 15 Department of Nutrition and Food Hygiene, Tongji Medical College, Huazhong University of Science and Technology, Wuhan, 430030 Hubei China; 16 Key Laboratory of Birth Defects and Related Diseases of Women and Children (Sichuan University), Ministry of Education, West China Second University Hospital, Sichuan University, Chengdu, 610041 Sichuan China; 17 NMPA Key Laboratory for Technical Research on Drug Products in Vitro and in Vivo Correlation, West China Second University Hospital, Sichuan University, Chengdu, 610041 Sichuan China; 18 Shuangliu Institute of Women’s and Children’s Health, Shuangliu Maternal and Child Health Hospital, Chengdu, 610200 Sichuan China

**Keywords:** Gestational diabetes mellitus, C-peptide, Pregnancy, Risk factor, Prediction

## Abstract

**Objective:**

To examine the association of early-pregnancy serum C-peptide with incident gestational diabetes mellitus (GDM) and the predictive ability of maternal C-peptide for GDM.

**Methods:**

A nested case–control study of 332 GDM cases and 664 controls was established based on the Tongji-Shuangliu Birth Cohort. The GDM cases and controls were matched at 1:2 on maternal age (± 3 years) and gestational age (± 4 weeks). Multivariable conditional logistic regression was applied to assess the association of C-peptide with risk of GDM. Partial Spearman’s correlation coefficients were estimated for the correlations between C-peptide and multiple metabolic biomarkers. C-statistics were calculated to assess the predictive ability of early-pregnancy C-peptide for GDM.

**Results:**

Of 996 pregnant women, median maternal age was 28.0 years old and median gestational age was 11.0 weeks. After adjustment for potential confounders, the odds ratio of GDM comparing the extreme quartiles of C-peptide was 2.28 (95% confidence interval, 1.43, 3.62; *P* for trend < 0.001). Partial correlation coefficients ranged between 0.07 and 0.77 for the correlations of C-peptide with fasting insulin, homeostatic model of insulin resistance, leptin, fasting blood glucose, triglycerides, glycosylated hemoglobin, waist–hip ratio, systolic blood pressure, and low-density lipoprotein cholesterol (*P* ≤ 0.025), and were − 0.11 and − 0.17 for high-density lipoprotein cholesterol and adiponectin (*P* < 0.001). Serum C-peptide slightly improved the predictive performance of the model with conventional predictive factors (0.66 vs. 0.63; *P* = 0.008).

**Conclusion:**

While the predictive value for subsequent GDM should be validated, early-pregnancy serum C-peptide may be positively associated with risk of GDM.

**Supplementary Information:**

The online version contains supplementary material available at 10.1186/s12986-022-00691-3.

## Background

Gestational diabetes mellitus (GDM) is defined as diabetes firstly diagnosed in the second or third trimester of pregnancy [1]. It is one of the most common metabolic disorders during pregnancy, with a prevalence of about 16.8% worldwide [2]. In China, the prevalence of GDM could reach up to 14.8% [3]. Since GDM is associated with a higher risk of type 2 diabetes in both the mothers [4] and offspring [5], its early detection and prevention has strong implications for the control of metabolic diseases.

C-peptide is a short 31-animo-acid protein that is secreted from pancreatic islet *β* cells into circulation in equimolar amounts with insulin. It is a constant biomarker to measure the *β* cell function because it has a longer half-life compared to insulin and is subject to negligible hepatic extraction before release to circulation [6]. Recent evidence indicates that C-peptide is an active peptide hormone with important physiologic functions and exerts metabolic effects [7]. While accumulated evidence suggests a link between C-peptide and type 2 diabetes [8, 9], fewer studies explored the effects of C-peptide on subsequent risk of GDM. Most previous studies on this topic utilized case–control or cross-sectional designs, and inherent limitations such as a lack of temporal associations made causal inference less reliable. To date, only three prospective studies assessed the association between early-pregnancy fasting plasma C-peptide and subsequent risk of GDM [10–12], and all these studies were conducted in European populations and reported an increased GDM risk associated with higher fasting C-peptide. Despite consistent results in previous studies, it is less clear whether the association varies across populations or exists among Chinese women. Understanding the role of C-peptide in GDM development might help to improve early intervention as well as prediction. However, despite a high prevalence of GDM in China, very little work has been undertaken to assess this association in Chinese women.

To expand our knowledge of the potential role of C-peptide in the development of GDM, in this prospective nested case–control study, we aimed to (1) examine the association of fasting serum C-peptide during early pregnancy with subsequent risk of GDM; (2) examine the correlations between C-peptide and major metabolic biomarkers in pregnant women; and (3) assess the ability of C-peptide for predicting GDM among Chinese women.

## Materials and methods

### Design and population

The nested case–control study was conducted in the Tongji-Shuangliu Birth Cohort (TSBC) [13], which was started from March 2017 in the Shuangliu Maternal and Child Health Hospital in Chengdu. Until June 2019, 6143 pregnant women were enrolled during their first prenatal examination (6–17 weeks of pregnancy). Women were included if they met the following criteria: (1) women aged 18–40 years with singleton pregnancy; and (2) gestational age less than 15 weeks. Participants were excluded if they (1) conceived the fetus using assisted reproductive technology, such as in-vitro fertilization and intrauterine insemination; (2) reported severe chronic disease or infectious disease like cancer, tuberculosis, and HIV infection; or (3) refused to sign the written informed consent or had no ability to complete the questionnaire independently. Structured questionnaires were administered at enrollment, and blood samples were obtained for future analyses. The original cohort study was approved by the Ethics Committee of Tongji Medical College, Huazhong University of Science and Technology, and informed consent was obtained from all participants.

### GDM diagnosis and matching to controls

GDM was diagnosed at 24–28 weeks of pregnancy according to the International Association of Diabetes in Pregnancy Study Groups criteria using the standard 75 g 2-h oral glucose tolerance test (OGTT): (1) fasting plasma glucose ≥ 5.1 mmol/L; and/or (2) 1-h plasma glucose ≥ 10.0 mmol/L; and/or (3) 2-h plasma glucose ≥ 8.5 mmol/L [14]. A total of 347 GDM women were diagnosed, of whom 14 did not provide sufficient blood samples for C-peptide measurements at enrollment and 1 had data missing for key covariates. We included 332 eligible GDM cases, and matched them individually to 664 pregnant women with normal glucose tolerance at 1:2 on maternal age (± 3 years) and gestational age (± 4 weeks) (Additional file 1: Figure S1).

### Measurement of serum C-peptide and other biomarkers

Measurement of metabolic biomarkers were conducted using fasting blood samples collected at enrollment. Serum C-peptide, insulin, and leptin were measured using the Metabolic Group 1 (hu) Singleplex Assays on the Meso Scale Discovery (MSD) U-PLEX Metabolic Platform (MSD, Rockville, Maryland, US). The intra- and inter-assay coefficients of variation for C-peptide were 3.7% and 10.3%, separately. Fasting blood glucose (FBG) was measured using the Glucose Assay Kit (Sichuan Maccura Biotechnology, Chengdu, China) by the method of GOD-PAP (glucose oxidase-phenol and 4 aminophenazone). Glycosylated hemoglobin (HbA1c) was measured using a DCA Vantage Analyzer (Siemens Healthcare Diagnostics, Marburg, Hessen, Germany). Serum high-sensitivity C-reactive protein (hs-CRP) and adiponectin were tested using the R&D enzyme-linked immunosorbent assays (R&D Systems, Minneapolis, Minnesota, US). Total cholesterol, triglycerides (TG), high-density lipoprotein cholesterol (HDL-C), and low-density lipoprotein cholesterol (LDL-C) were measured via the Mindray BS-200 chemistry Analyzer (Mindray Medical International, Shenzhen, China). The homeostatic model of insulin resistance (HOMA-IR) was used to estimate insulin resistance and calculated based on the following formula: HOMA-IR = fasting blood glucose (mmol/L) × fasting insulin (mIU/L)/22.5 [15]. Missing values for FBG (n = 11), HbA1c (n = 17), and serum lipids (n = 5) were imputed using median values by GDM status in the study.

### Measurement of covariates

Data of sociodemographic information, history of disease and reproduction, and lifestyle and behaviors were obtained via questionnaire interviews at enrollment. Anthropometric measurements were conducted at enrollment per standard protocols. Pre-pregnancy body mass index (BMI) was calculated according to the formula: BMI = weight (kilogram)/height^2^ (meter), in which pre-pregnancy weight was self-reported. Waist–hip ratio (WHR) was defined as waistline (cm) divided by hipline (cm). Education level was categorized according to years of education: ≤ 12 years and > 12 years. Smoking status and alcohol consumption were both categorized as never, former, and current. Blood pressure was measured twice using Omron electronic sphygmomanometer (Omron, Kyoto, Japan), and the average value was calculated. Physical activity in metabolic equivalent of task (MET)-hours per week was evaluated using the Chinese version of the Pregnancy Physical Activity Questionnaire [16], which has been validated among Chinese pregnant women [17]. Parity was classified into 0 and ≥ 1. Parental history of diabetes and history of GDM were both defined as yes and no.

### Statistical analysis

For descriptive analyses, continuous variables were reported as mean and standard deviation (SD) or as median and interquartile range (IQR), and categorical variables as frequency and percentage. Baseline characteristics among C-peptide quartile groups were compared using Kruskal–Wallis test or analysis of variance for continuous variables and chi-square test for categorical variables. In addition, baseline characteristics between GDM cases and controls were compared by univariable conditional logistic regression.

Partial Spearman regression was used to examine the relationship of C-peptide with multiple metabolic biomarkers including WHR, blood pressure, FBG, fasting insulin, HOMA-IR, HbA1c, total cholesterol, TG, LDL-C, HDL-C, hs-CRP, adiponectin, and leptin in early pregnancy among all included pregnant women, with adjustment for maternal age, gestational age, education level, smoking status, alcohol consumption, physical activity, pre-pregnancy BMI, parental history of diabetes, history of GDM, parity, and GDM status.

Multivariable conditional logistic regression models were used to estimate odds ratios (ORs) and their 95% confidence intervals (CIs) between early-pregnancy serum C-peptide and risk of GDM. C-peptide was assessed as a categorical variable (quartiles based on the concentration among the control group), and as continuous variables (on the natural log scale and for each 1-SD change). Covariates were sequentially adjusted for in two models: maternal age (continuous, years), gestational age (continuous, weeks), and education level (≤ 12 years and > 12 years) in Model 1; additionally, smoking status (never, former, and current), alcohol consumption (never, former, and current), physical activity (continuous, MET-hours per week), pre-pregnancy BMI (continuous, kg/m^2^), parental history of diabetes (yes and no), history of GDM (yes and no), and parity (0 and ≥ 1) in Model 2. In sensitivity analyses, we separately adjusted for insulin (continuous, uIU/mL), HOMA-IR (continuous), and leptin (continuous, ng/mL) in multivariable conditional logistic regression model due to their stronger correlations to C-peptide.

*P* values for trend were estimated by modeling the median value of each C-peptide quartile as a continuous variable. We used restricted cubic splines with five knots at the 5th (reference), 27.5th, 50th, 72.5th, and 95th centiles to model the non-linear association between C-peptide and GDM. To investigate whether the association was modified by the baseline characteristics, we conducted subgroup analyses by maternal age (< 30 and ≥ 30 years), pre-pregnancy BMI (< 24.0 and ≥ 24.0 kg/m^2^), and parental history of diabetes (yes and no). Interactions (effect modifications) were assessed via the likelihood ratio test by adding an interaction term of a stratifying variable and C-peptide.

We calculated C-statistics based on logistic regression models to assess the predictive ability of early-pregnancy C-peptide for GDM. Four models were established in our analyses: Model 1 included conventional predictive factors for GDM including maternal age, gestational age, pre-pregnancy BMI, physical activity, parental history of DM, and history of GDM; Model 2 included conventional predictive factors and C-peptide; Model 3 included conventional predictive factors and FBG; Model 4 included conventional predictive factors, FBG, and C-peptide. To compare the discriminative performance, the Delong test was used to compare the C-statistics. Moreover, we used net reclassification improvement (NRI) [18] and integrated discrimination improvement (IDI) [19] statistics to measure the utility of C-peptide in GDM prediction.

Data analyses were performed by STATA 15.0 (Stata Corporation, College Station, TX, US). Partial Spearman’s correlation coefficients were visualized by GraphPad Prism 8 (GraphPad Software Inc., San Diego, CA, USA). NRI and IDI were calculated by comparison of predictive models using SAS 9.4 (SAS Institute, Cary, NC, USA). Two-sided *P* < 0.05 was considered to indicate statistical significance.

## Results

### Baseline characteristics of participants

Of 996 pregnant women, the median maternal age (IQR) was 28.0 (25.0–30.0) years and median gestational age was 11.0 (9.0–12.0) weeks. Women with higher C-peptide levels showed higher pre-pregnancy BMI, WHR, blood pressure, FBG, fasting insulin, HOMA-IR, HbA1c, TG, LDL-C, and lower HDL-C. In addition, women with higher C-peptide levels were more likely to be multiparous, poorly educated, and have history of GDM (Table 1). Comparisons of baseline characteristics between GDM cases and controls are presented in Additional file 1: Table S1.

**Table 1:** Baseline characteristics of 996 study participants

Characteristics	Total	Quartiles of C-peptide				*P* values^a^
		Quartile 1	Quartile 2	Quartile 3	Quartile 4	
Maternal age, years	28.0 (25.0–30.0)	27.0 (25.0–30.0)	27.0 (25.0–30.0)	28.0 (25.0–30.0)	28.0 (25.0–31.0)	0.483
Gestational age, weeks	11.0 (9.0–12.0)	11.0 (9.0–12.0)	11.0 (9.0–12.0)	11.0 (9.0–12.0)	10.0 (8.0–12.0)	0.007
Education > 12 years, n (%)	399 (40.1)	94 (43.7)	94 (42.0)	94 (37.8)	117 (38.0)	0.450
Parity ≥ 1, n (%)	502 (50.4)	93 (43.3)	107 (47.8)	128 (51.4)	174 (56.5)	0.021
Smoking status, n (%)						0.538
Current	16 (1.6)	2 (0.9)	4 (1.8)	4 (1.6)	6 (2.0)	
Former	42 (4.2)	6 (2.8)	14 6.3)	8 (3.2)	14 (4.6)	
Never	938 (94.2)	207 (96.3)	206 (92.0)	237 (95.2)	288 (93.5)	
Alcohol consumption, n (%)						0.149
Current	3 (0.3)	1 (0.5)	0	0	2 (0.7)	
Former	188 (18.9)	32 (14.9)	54 (24.1)	43 (17.3)	59 (19.2)	
Never	805 (80.8)	182 (84.7)	170 (75.9)	206 (82.7)	247 (80.2)	
Physical activity, MET-h∙week-1	116.5 (70.5–171.0)	126.7 (77.3–183.7)	121.6 (75.5–171.9)	123.6 (68.1–171.0)	106.8 (63.7–161.1)	0.078
Pre-pregnancy BMI, kg/m^2^	21.0 (19.2–23.0)	19.7 (18.3–21.2)	20.3 (18.8–21.6)	21.0 (19.3–22.7)	22.8 (21.1–25.3)	< 0.001
WHR	0.8 (0.8–0.9)	0.8 (0.8–0.8)	0.8 (0.8–0.9)	0.8 (0.8–0.9)	0.8 (0.8–0.9)	< 0.001
Systolic blood pressure, mmHg	109.0 (103.5–116.0)	107.0 (101.5–113.5)	108.0 (102.5–114.5)	109.0 (104.5–115.5)	112.0 (105.5–119.0)	< 0.001
Diastolic blood pressure, mmHg	74.0 (68.5–79.0)	73.0 (67.0–77.0)	74.0 (68.0–78.0)	74.0 (68.5–79.5)	75.5 (70.0–81.3)	< 0.001
C-peptide, ng/mL	0.92 (0.73–1.20)	0.58 (0.51–0.64)	0.78 (0.74–0.82)	0.98 (0.92–1.05)	1.39 (1.24–1.71)	N/A
Fasting blood glucose, mmol/L	4.4 (4.2–4.7)	4.2 (4.0–4.5)	4.4 (4.1–4.6)	4.4 (4.2–4.7)	4.6 (4.3–4.9)	< 0.001
Insulin, uIU/mL	8.0 (5.5–11.5)	4.7 (3.6–5.5)	6.4 (5.4–7.6)	8.6 (7.4–10.5)	13.1 (10.3–17.0)	< 0.001
HOMA-IR	1.6 (1.1–2.3)	0.9 (0.7–1.0)	1.2 (1.0–1.5)	1.7 (1.4–2.1)	2.7 (2.1–3.5)	< 0.001
HbA1c, %^b^	5.1 ± 0.3	5.0 ± 0.3	5.0 ± 0.2	5.1 ± 0.2	5.1 ± 0.3	< 0.001
Total cholesterol, mmol/L	5.1 (4.4–5.7)	5.1 (4.4–5.7)	5.1 (4.4–5.6)	5.0 (4.3–5.6)	5.2 (4.5–5.8)	0.269
TG, mmol/L	1.5 (1.1–1.9)	1.3 (1.0–1.6)	1.4 (1.0–1.8)	1.4 (1.1–1.9)	1.7 (1.2–2.3)	< 0.001
LDL-C, mmol/L	1.7 (1.4–2.0)	1.6 (1.3–1.9)	1.6 (1.4–1.9)	1.6 (1.4–1.9)	1.7 (1.5–2.1)	< 0.001
HDL-C, mmol/L	1.4 (1.2–1.6)	1.5 (1.4–1.7)	1.5 (1.3–1.7)	1.4 (1.2–1.6)	1.3 (1.2–1.5)	< 0.001
Parental history of DM, n (%)	78 (7.8)	11 (5.1)	15 (6.7)	19 (7.6)	33 (10.7)	0.091
History of GDM, n (%)	27 (2.7)	1 (0.5)	3 (1.3)	6 (2.4)	17 (5.5)	0.001

Data are presented as mean (standard deviation) or median (interquartile range) for continuous variables, and frequency (percentages) for categorical variables

*BMI* body mass index, *DM* diabetes mellitus, *GDM* gestational diabetes mellitus, *HbA1c* glycosylated hemoglobin, *HDL-C* high-density lipoprotein cholesterol, *HOMA-IR* homoeostatic model assessment‐insulin resistance, *LDL-C* low-density lipoprotein cholesterol, *N/A* not applicable, *TG* triglycerides, *WHR* waist–hip ratio

^a^*P* values were calculated by Kruskal–Wallis Test for continuous variables and chi-square test for categorical variables

^b^*P* values were calculated by analysis of variance

### Correlations between serum C-peptide and multiple metabolic biomarkers in early pregnancy

**Fig. 1:**
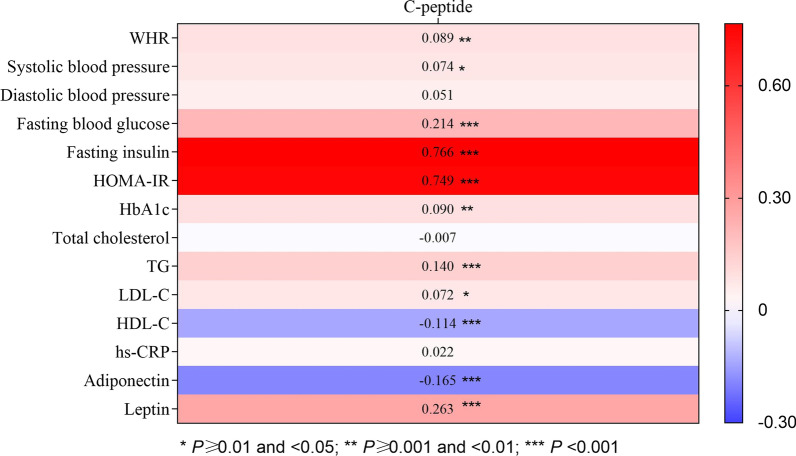
Partial spearman correlations between baseline metabolic traits and C-peptide. *HbA1c* glycosylated hemoglobin, *HDL-C* high-density lipoprotein cholesterol, *HOMA-IR* homoeostatic model assessment‐insulin resistance, *hs-CRP* high-sensitivity C-reactive protein, *LDL-C* low-density lipoprotein cholesterol, *TG* triglycerides, *WHR* waist–hip ratio. *P* values were calculated using partial spearman regression with adjustment for maternal age, gestational age, education, parity, smoking status, alcohol consumption, physical activity, pre-pregnancy BMI, family history of diabetes, history of GDM, and GDM status. There were positive correlations of C-peptide with fasting insulin, homeostatic model of insulin resistance, leptin, fasting blood glucose, triglycerides, glycosylated hemoglobin, waist–hip ratio, systolic blood pressure, and low-density lipoprotein cholesterol, and negative correlations with high-density lipoprotein cholesterol and adiponectin, demonstrating an adverse metabolic profile associated with C-peptide

Among 996 pregnant women, we found positive correlations of serum C-peptide with maternal fasting insulin (*β* = 0.77; *P* < 0.001), HOMA-IR (*β* = 0.75; *P* < 0.001), leptin (*β* = 0.26; *P* < 0.001), FBG (*β* = 0.21; *P* < 0.001), TG (*β* = 0.14; *P* < 0.001), HbA1c (*β* = 0.09; *P* = 0.005), WHR (*β* = 0.09; *P* = 0.006), systolic blood pressure (*β* = 0.07; *P* = 0.021), and LDL-C (*β* = 0.07; *P* = 0.025). In contrast, high levels of serum C-peptide were correlated with lower HDL-C (*β* = -0.11; *P* < 0.001) and adiponectin (*β* = -0.17; *P* < 0.001; Fig. 1).

### Association between early-pregnancy C-peptide and risk of GDM

Pregnant women with elevated early-pregnancy C-peptide levels showed a higher risk of GDM, with an OR (95% CI) of 2.28 (1.43, 3.62) for the extreme-quartile comparison, after adjustment for maternal age, gestational age, education level, smoking status, alcohol consumption, physical activity, pre-pregnancy BMI, history of parental diabetes, history of GDM, and parity. ORs of GDM were 2.64 (1.76, 3.96) and 1.33 (1.16, 1.54) for each 1 log ng/mL and each 1 SD ng/mL increase of C-peptide, respectively. There was a linear trend in the association of C-peptide with risk of GDM (*P* for trend < 0.001; Table 2). Modeling with restricted cubic splines showed little evidence for a non-linear relationship between C-peptide and risk of GDM (*P* for overall association < 0.001; *P* for non-linearity = 0.082; Additional file 1: Figure S2).

**Table 2:** Association between early-pregnancy C-peptide and risk for GDM

C-peptide	Case/Sample	Model 1^a^: OR (95% CI)	Model 2^b^: OR (95% CI)
Quartile 1	49/215	1.00	1.00
Quartile 2	58/224	1.12 (0.71, 1.76)	1.06 (0.66, 1.69)
Quartile 3	83/249	1.76 (1.15, 2.67)	1.52 (0.97, 2.36)
Quartile 4	142/308	3.06 (2.03, 4.61)	2.28 (1.43, 3.62)
Per 1 log ng/mL	332/996	3.21 (2.25, 4.58)	2.64 (1.76, 3.96)
Per 1 SD ng/mL	332/996	1.45 (1.27, 1.66)	1.33 (1.16, 1.54)
*P* for trend^c^	332/996	< 0.001	< 0.001

*CI* confidence interval, *GDM* gestational diabetes mellitus, *OR* odds ratio, *SD* standard deviation

^a^Adjusted for maternal age (continuous, years), gestational age (continuous, weeks), and education level (≤ 12 years and > 12 years)

^b^Adjusted for smoking status (never, former, and current), alcohol consumption (never, former, and current), physical activity (continuous, MET-h∙week-1), pre-pregnancy BMI (continuous, kg/m^2^), parental history of diabetes (yes and no), history of gestational diabetes (yes and no), parity (0 and ≥ 1), and variables adjusted for in Model 1

^c^Linear trend was estimated by replacing the values of C-peptide by the median value of each quartile, and modeling C-peptide as continuous variable in Model 2

After additional adjustment for insulin, the association between early-pregnancy C-peptide and GDM was attenuated (OR, 1.94; 95% CI 1.13, 3.34 for the extreme-quartile comparison). ORs of GDM were 3.45 (1.79, 6.65) and 1.47 (1.12, 1.94) for 1 log ng/mL increase and 1-SD increase of C-peptide, respectively (Additional file 1: Table S2). In addition, when we separately controlled for HOMA-IR (1.72; 1.00, 2.97 for the extreme-quartile comparison) and leptin (2.11; 1.30, 3.42) in multivariable conditional logistic regression models, pregnant women with higher early-pregnancy serum C-peptide levels still showed increased risks of GDM (Additional file 1: Table S2). In subgroup analyses, no significant interactions were observed between C-peptide and maternal age, pre-pregnancy BMI, or parental history of diabetes for GDM risk (all *P* for interaction ≥ 0.089; Additional file 1: Table S3).

### Performance of early-pregnancy C-peptide in GDM prediction

**Fig. 2:**
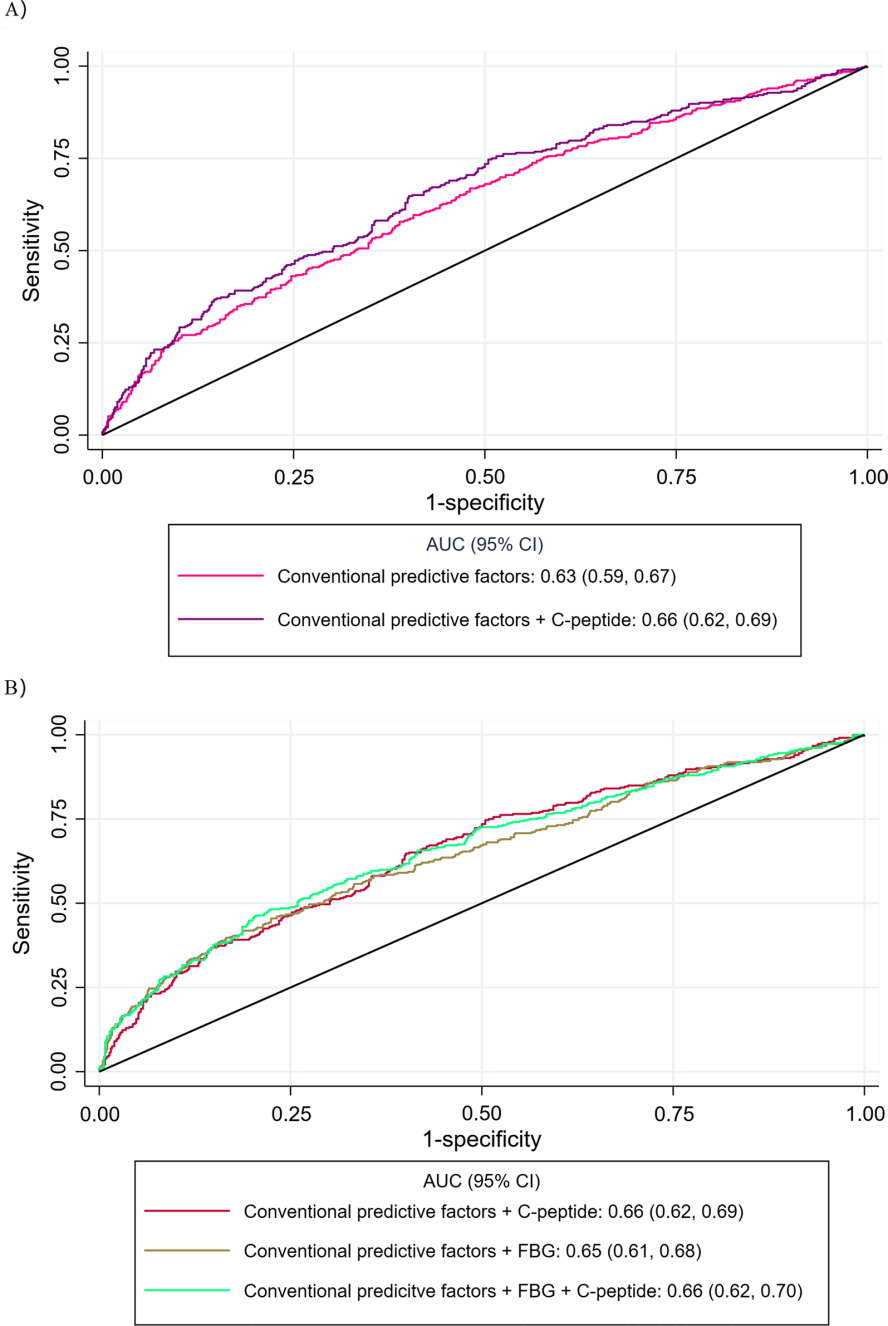
Receiver operator characteristic curves for early-pregnancy fasting biomarkers of glucose metabolism in gestational diabetes mellitus prediction. (**A**) Comparation of models based on conventional predictive factors and conventional predictive factors plus C-peptide (Difference: 0.03; *P* = 0.008); (**B**) Comparation of models with (1) conventional predictive factors and C-peptide, (2) conventional predictive factors and FBG, (3) conventional predictive factors, FBG, and C-peptide. Conventional predictive factors included maternal age, gestational age, pre-pregnancy body mass index, physical activity, parental history of diabetes mellitus, and history of GDM. *AUC* area under receiver operator characteristic curve, *CI* confidence interval, *FBG* fasting blood glucose, *GDM* gestational diabetes mellitus

For the GDM prediction analyses, the C-statistic for the base model with conventional predictive factors was 0.63 (0.59, 0.67). Adding C-peptide to the base model only yielded a slight improvement of 0.03 in the C-statistic (*P* = 0.008), while no significant change was observed when FBG was added (*P* = 0.240; Fig. 2 and Additional file 1: Table S4). Meanwhile, we observed a mild increment of NRI (C-peptide: 19.6, *P* = 0.036; FBG: 16.0, *P* = 0.018) and IDI (C-peptide: 0.018, *P* < 0.001; FBG: 0.023, *P* < 0.001; Additional file 1: Table S4) in two models. Compared to the model with conventional predictive factors and FBG, the model with conventional predictive factors and C-peptide showed a similar predictive ability (*P* = 0.412; Fig. 2 and Additional file 1: Table S4). In addition, adding C-peptide to the model with conventional predictive factors and FBG yielded a mild increase of 0.01 in the C-statistic (0.66 vs. 0.65; *P* = 0.021). For the same comparison, we only noted mild NRI (*P* = 0.003) and IDI (*P* = 0.005; Additional file 1: Table S4).

## Discussion

Our study documented positive associations between early-pregnancy serum C-peptide and risk of developing GDM among pregnant women in China. This finding was also supported by significant correlations between C-peptide and metabolic biomarkers in pregnant women. C-peptide alone was at least comparable to FBG when added to conventional predictive factors for predicting GDM. Our findings suggest that early-pregnancy C-peptide could be an important risk factor for GDM, although the predictive value for subsequent GDM needs to be confirmed in future large prospective studies.

In our study, higher C-peptide was linearly associated with incident GDM in Chinese pregnant women. Consistent with our finding, in the Omega study among 804 Swedish pregnant women free of pre-existing diabetes, the risk of GDM among women with C-peptide ≥ 3.00 ng/mL showed a 2.28-fold increase risk compared to those with a concentration < 1.45 ng/mL [10]. Meanwhile, the Camden study among 574 Austrian women showed that higher levels of fasting C-peptide before 16 weeks of pregnancy were associated with an increased risk of GDM, and the adjusted OR was 1.85 per 1 ng/mL increase [11]. In addition, in another prospective cohort study among 1,368 British pregnant women, those with C-peptide ≥ 0.54 nmol/L experienced a 4.43-fold higher risk of subsequent GDM [12]. A small case–control study in 82 pregnant women (42 GDM cases and 40 cases of normal pregnant women) also found elevated C-peptide in the GDM group in the third trimester in China [20]. The slight differences in the strength of association between previous studies and ours might be attributable to variations in study populations, study designs, different sample size, diagnosis criteria of GDM, gestational age of C-peptide measurement, and statistical modelling. The evidence based on clinical and experimental studies demonstrates that C-peptide could stimulate glucose transport [21], dampen the metabolic effects of insulin at high serum concentrations [22], promote lipids accumulation in adipocytes [23] and vascular walls [24], and accelerate central obesity [25]. The mechanistic evidence is supported by our finding that serum C-peptide was correlated positively with metabolic profiles including high levels of FBG, fasting insulin, HOMA-IR, HbA1c, TG, LDL-C, leptin, and WHR, but negatively with HDL-C and adiponectin. Our study has been the first prospective one to address this topic among Chinese women and further consolidate previous findings.

Since the diagnosis of GDM is often recommended for the late second or early third trimester of pregnancy according to established guidelines [14], only a small window of intervention is possible to minimize the adverse effect of GDM. A number of studies predicted the development of GDM using basic characteristics and easily available clinical biomarkers [26, 27], which might facilitate recognition of women with high risk of subsequent GDM and targeted intervention for GDM at an earlier stage. Lamain–de Ruiter et al. [28] reviewed prediction models for the risk of GDM, and these models were mostly based on traditional clinical risk factors and showed limited discriminative capability. In our study, early-pregnancy C-peptide compared well with FBG when added to conventional predictive factors for predicting GDM, and slightly improved the prediction based on conventional predictive factors and FBG. One predictive model based on both clinical and biochemical predictors including fasting plasma glucose, TG, and HbA1c at early pregnancy had an C-statistic of 0.72, which was slightly higher than the van Leeuwen [29] and the Teede [30] prediction models just based on clinical factors in the same population [31]. Recently, a GDM prediction model in Chinese population based on clinical and biochemical predictors also achieved effective discriminate power (C-statistic = 0.77) [26]. The prediction performance of our model was slightly weakener compared to above-mentioned ones, which could be attributed to differences in the study populations, methods of modeling, predictors for modeling, gestational age of predictors measurement, and diagnosed criteria for GDM. Of note, inclusion of biomarkers of glucose metabolism contributed to the optimization of GDM prediction models. However, due to the under-recognition of C-peptide in glucose metabolism, few studies assessed the accuracy of C-peptide prediction for GDM. One conducted in Vienna reported that C-peptide performed well for prediction of GDM, especially for GDM with a need of pharmacotherapy (C-statistic = 82.2%) [11]. Thus, the potential predictive ability of C-peptide in early pregnancy for subsequent GDM should be further assessed in future studies.

Although the mechanisms of GDM were not yet fully defined, C-peptide might be involved in the GDM development by the pathways of insulin resistance, lipid metabolism, and inflammation. First, the metabolic effects of insulin could be enhanced by C-peptide at low hormone concentrations and reduced at high concentrations [22]. Thus, high C-peptide level is considered a marker of decreased insulin sensitivity, which is one of the main metabolic abnormalities underlying GDM [32]. Second, C-peptide has been reported to promote the lipid accumulation via the pathway of stimulating peroxisome proliferator-activated receptor-γ (PPAR-γ). C-peptide could regulate the expression of PPAR-γ regulated genes involved in metabolic control and inflammation [33]. PPAR-γ, as a requisite transcription factor in the differentiation of adipose tissue, could enhance the lipids accumulation in adipocyte and then lead to lipid metabolism disorder [23]. Of note, dyslipidemia such as increased triglycerides was reported to associate with insulin resistance and damaged *β* cell function independent of overweight or obesity status [34]. Third, C-peptide was reported to show proinflammatory effects in different body tissues. Animal studies demonstrated that elevated C-peptide promoted inflammatory cell infiltration in ApoE-deficient mice [24]. Moreover, chronic subclinical inflammation is considered a part of insulin resistance syndrome, which has a central role in the development of GDM [35]. Our finding could have key clinical implications. C-peptide might be a useful biomarker for GDM in early pregnancy, and could be screened routinely in addition to other glucose metabolism biomarkers. Women with elevated C-peptide in their early pregnancy would need to be monitored for potential risk of future GDM. However, future basic and clinical studies are warranted to elucidate the mechanisms by which elevated early-pregnancy C-peptide increases the risk of GDM.

To our knowledge, this has been the first prospective study to examine the association between early-pregnancy C-peptide and the risk of subsequent GDM among Chinese pregnant women. The prospective design allowed us to better elucidate the temporal relationship. Well-matched controls guaranteed the comparability of baseline characteristics of cases and controls to generate unbiased estimates. Despite the strengths mentioned, certain limitations must be acknowledged. First, how the dynamics of C-peptide levels during pregnancy affect risk of GDM could not be addressed because C-peptide was only measured once in our study. Of note, mild insulin resistance develops physiologically to adapt to fetal growth [36], so future studies could be directed to the impact of C-peptide changes on GDM across different trimesters. Second, our study was not aimed to reveal the mechanisms of C-peptide in the development of GDM, so the prospective association should not be interpreted as an indication of a causal link between C-peptide and GDM. Third, our study had a moderate sample size and the study population was from a district of western China, which restricts the generalizability of our findings. The identified association between C-peptide and risk of GDM and the predictive model should be further validated in other large populations.

In conclusion, we observed a positive association between higher early-pregnancy C-peptide and increased risk of subsequent GDM among Chinese pregnant women. C-peptide was also correlated with unfavorable metabolic profiles in Chinese pregnant women. Our findings highlight the role of C-peptide in the development of GDM and the potential of using early-pregnancy C-peptide as a routine biomarker for predicting the risk of GDM.

## Supplementary Information

**Additional file 1**. **Table S1**: Baseline characteristics of GDM patients and matched controls. **Table S2**: Association between early-pregnancy C-peptide and risk for GDM in three sensitivity analyses. **Table S3**: Association between early-pregnancy C-peptide and GDM risk in subgroup analyses. **Table S4**: Summary statistics to assess the early-pregnancy C-peptide in predicting gestational diabetes mellitus. **Figure S1**: Flowchart of participants selection in the nested case-control study. **Figure S2**: Restricted cubic splines-based modeling for the association between early-pregnancy serum C-peptide and GDM.

## Data Availability

The datasets analyzed during the current study are not publicly available but are available from the corresponding author on reasonable request.
