# Supplementary material for: Association between early-pregnancy serum C-peptide and risk of gestational diabetes mellitus: a nested case–control study among Chinese women
Source: Nutr Metab (Lond). 2022 Aug 22;19:56. doi: 10.1186/s12986-022-00691-3 (PMC9396763; doi:10.1186/s12986-022-00691-3)
Supplement: Supplementary file 1 — Additional file 1. Table S1: Baseline characteristics of GDM patients and matched controls. Table S2: Association between early-pregnancy C-peptide and risk for GDM in three sensitivity analyses. Table S3: Association between early-pregnancy C-peptide and GDM risk in subgroup analyses. Table S4: Summary statistics to assess the early-pregnancy C-peptide in predicting gestational diabetes mellitus. Figure S1: Flowchart of participants selection in the nested case-control study. Figure S2: Restricted cubic splines-based modeling for the association between early-pregnancy serum C-peptide and GDM. [file 12986_2022_691_MOESM1_ESM.docx]

**Additional file 1**

**Association between early-pregnancy serum C-peptide and risk of gestational diabetes mellitus: a nested case-control study among Chinese women**

Xue Yang ^*^, Yi Ye^*^, Yi Wang, Ping Wu, Qi Lu, Yan Liu, Jiaying Yuan, Xingyue Song, Shijiao Yan, Xiaorong Q, Yi-Xin Wang, Ying Wen, Gang Liu, Chuanzhu Lv, Chun-Xia Yang, An Pan, Jianli Zhang^#^, Xiong-Fei Pan ^#^

Table S1 Baseline characteristics of GDM patients and matched controls.

Table S2 Association between early-pregnancy C-peptide and risk for GDM in three sensitivity analyses.

Table S3 Association between early-pregnancy C-peptide and GDM risk in subgroup analyses.

Table S4 Summary statistics to assess the early-pregnancy C-peptide in predicting gestational diabetes mellitus.

Figure S1 Flowchart of participants selection in the nested case-control study.

Figure S2 Restricted cubic splines-based modeling for the association between early-pregnancy serum C-peptide and GDM.

**Table S1 Baseline characteristics of GDM cases and matched controls.**

|  | GDM cases | Non-GDM controls | *P* values ^a^ |
| --- | --- | --- | --- |
| Maternal age, years | 28.0 (25.0-31.0) | 28.0 (25.0-30.0) | - |
| Gestational age, weeks | 11.0 (9.0-12.0) | 11.0 (9.0-12.0) | - |
| Education, n (%) |  |  | 0.053 |
| ≤12 years | 213 (64.2) | 384 (57.8) |  |
| >12 years | 119 (35.8) | 280 (42.2) |  |
| Parity, n (%) |  |  | 0.505 |
| 0 | 169 (50.9) | 325 (49.0) |  |
| ≥1 | 163 (49.1) | 339 (51.0) |  |
| Smoking status, n (%) |  |  | 0.383 |
| Current | 6 (1.8) | 10 (1.5) |  |
| Former | 18 (5.4) | 24 (3.6) |  |
| Never | 308 (92.8) | 630 (94.9) |  |
| Alcohol consumption, n (%) |  |  | 0.218 |
| Current | 1 (0.3) | 2 (0.3) |  |
| Former | 73 (22.0) | 115 (17.3) |  |
| Never | 258 (77.7) | 547 (82.4) |  |
| Physical activity, MET-h∙week-1 | 109.7 (62.3-168.5) | 119.7 (73.8-173.4) | 0.047 |
| Pre-pregnancy BMI, kg/m^2^ | 21.6 (20.0-24.0) | 20.8 (19.0-22.6) | <0.001 |
| WHR | 0.8 (0.8-0.9) | 0.8 (0.8-0.9) | <0.001 |
| Systolic blood pressure, mmHg | 111.3 (105.5-118.5) | 108.5 (102.5-114.5) | <0.001 |
| Diastolic blood pressure, mmHg | 75.0 (69.0-81.0) | 74.0 (68.0-78.0) | 0.001 |
| Fasting blood glucose, mmol/L | 4.5 (4.2-4.8) | 4.4 (4.1-4.6) | <0.001 |
| C-peptide, ng/mL | 1.1 (0.8-1.4) | 0.9 (0.7-1.1) | <0.001 |
| Insulin, uIU/mL | 9.1 (6.4-13.1) | 7.4 (5.4-10.6) | <0.001 |
| HOMA-IR | 1.8 (1.3-2.7) | 1.4 (1.0-2.1) | <0.001 |
| HbA1c, % | 5.2±0.3 | 5.0±0.2 | <0.001 |
| Total cholesterol, mmol/L | 5.2 (4.5-5.8) | 5.0 (4.4-5.6) | 0.029 |
| TG, mmol/L | 1.5 (1.1-2.0) | 1.4 (1.1-1.9) | 0.002 |
| LDL-C, mmol/L | 1.7 (1.5, 2.1) | 1.6 (1.4-1.9) | <0.001 |
| HDL-C, mmol/L | 1.4 (1.2-1.6) | 1.5 (1.3-1.7) | 0.011 |
| Parental history of DM, n (%) | 38 (11.5) | 40 (6.0) | 0.003 |
| History of GDM, n (%) | 19 (5.7) | 8 (1.2) | <0.001 |

^a^ *P* values were calculated by univariable conditional logistic regression.

Data were presented as mean (IQR) for continuous variables and frequency (%) for categorical variables.

Abbreviations: BMI, body mass index; DM, diabetes mellitus; GDM, gestational diabetes mellitus; HbA1c, glycosylated hemoglobin; HDL-C, high-density lipoprotein cholesterol; HOMA-IR, Homoeostatic model assessment‐insulin resistance; IQR, interquartile range; LDL-C, low-density lipoprotein cholesterol; TG, triglycerides; WHR, waist-hip ratio

**Table S2 Association between** **early-pregnancy C-peptide and risk for GDM in three sensitivity analyses**

| C-peptide | Case/Sample | OR (95% CI) ^a^ | OR (95% CI) ^b^ | OR (95% CI) ^c^ |
| --- | --- | --- | --- | --- |
| Quartile 1 | 49/215 | 1.00 | 1.00 | 1.00 |
| Quartile 2 | 58/224 | 1.02 (0.64, 1.64) | 1.00 (0.62, 1.61) | 1.03 (0.64, 1.65) |
| Quartile 3 | 83/249 | 1..42 (0.91, 2.25) | 1.36 (0.86, 2.14) | 1.47 (0.94, 2.29) |
| Quartile 4 | 142/308 | 1.94 (1.13, 3.34) | 1.72 (1.00, 2.97) | 2.11 (1.30, 3.42) |
| Per 1 log ng/mL | 332/996 | 3.45 (1.79, 6.65) | 2.54 (1.35, 4.77) | 2.52 (1.64, 3.86) |
| Per 1 SD ng/mL | 332/996 | 1.47 (1.12, 1.94) | 1.26 (0.99, 1.61) | 1.30 (1.13, 1.51) |
| *P* for trend ^d^ | 332/996 | 0.008 | 0.027 | 0.001 |

CI, confidence interval; GDM, gestational diabetes mellitus; OR, odds ratio; SD, standard deviation.

^a^ Adjusted for maternal age (continuous, year), gestational age (continuous, week), education level (≤12 years and >12 years), smoking status (never, former, and current), alcohol consumption (never, former, and current), physical activity (continuous, MET-h∙week-1), baseline BMI (continuous, kg/m^2^), parental history of diabetes (yes and no), history of gestational diabetes mellitus (yes and no), parity (0 and ≥1), and fasting insulin (continuous, uIU/mL).

^b^ Adjusted for maternal age, gestational age, education level, smoking status, alcohol consumption, physical activity, pre-pregnancy BMI, parental history of diabetes, history of gestational diabetes mellitus, parity, and HOMA-IR (continuous).

^c^ Adjusted for maternal age, gestational age, education level, smoking status, alcohol consumption, physical activity, pre-pregnancy BMI, parental history of diabetes, history of gestational diabetes mellitus, parity, and leptin (continuous, ng/mL).

^d^ Linear trend was estimated by replacing the values of C-peptide by the median value of each quartile, and modeling C-peptide as a continuous variable.

**Table S3 Association between early-pregnancy C-peptide and GDM risk in subgroup analyses**

| C-peptide | Case/sample | Model 1 ^a^:  OR (95% CI) | Model 2 ^b^:  OR (95% CI) | *P* for interaction ^c^ |
| --- | --- | --- | --- | --- |
| **Maternal age, years** |  |  |  | 0.521 |
| <30 |  |  |  |  |
| Quartile 1 | 33/149 | 1.00 | 1.00 |  |
| Quartile 2 | 45/154 | 1.46 (0.89, 2.46) | 1.37 (0.80, 2.34) |  |
| Quartile 3 | 53/168 | 1.63 (0.98, 2.70) | 1.51 (0.89, 2.56) |  |
| Quartile 4 | 90/199 | 2.95 (1.82, 4.78) | 2.35 (1.36, 4.04) |  |
| ≥30 |  |  |  |  |
| Quartile 1 | 16/66 | 1.00 | 1.00 |  |
| Quartile 2 | 13/70 | 0.68 (0.30, 1.56) | 0.66 (0.28, 1.56) |  |
| Quartile 3 | 30/81 | 1.77 (0.85, 3.68) | 1.49 (0.70, 3.21) |  |
| Quartile 4 | 52/109 | 2.70 (1.36, 5.36) | 1.96 (0.91, 4.23) |  |
| **BMI, kg/m^2^** |  |  |  | 0.592 |
| BMI <24.0 |  |  |  |  |
| Quartile 1 | 48/208 | 1.00 | 1.00 |  |
| Quartile 2 | 55/204 | 1.23 (0.78, 1.92) | 1.12 (0.71, 1.77) |  |
| Quartile 3 | 70/214 | 1.61 (1.04, 2.49) | 1.39 (0.89, 2.18) |  |
| Quartile 4 | 75/185 | 2.29 (1.47, 3.58) | 2.78 (1.11, 2.86) |  |
| BMI ≥24.0 |  |  |  |  |
| Quartile 1 | 1/7 | 1.00 | 1.00 |  |
| Quartile 2 | 3/20 | 1.05 (0.09, 12.38) | 1.07 (0.09, 12.72) |  |
| Quartile 3 | 13/35 | 3.61 (0.38, 33.94) | 3.86 (0.40, 37.32) |  |
| Quartile 4 | 67/123 | 7.33 (0.84, 63.80) | 7.46 (0.83, 67.29) |  |
| **Parental history of diabetes** |  |  |  | 0.089 |
| No |  |  |  |  |
| Quartile 1 | 47/204 | 1.00 | 1.00 |  |
| Quartile 2 | 53/209 | 1.12 (0.72, 1.77) | 1.06 (0.67, 1.68) |  |
| Quartile 3 | 74/230 | 1.56 (1.02, 2.40) | 1.40 (0.90, 2.29) |  |
| Quartile 4 | 120/275 | 2.55 (1.70, 3.84) | 1.98 (1.26, 3.11) |  |
| Yes |  |  |  |  |
| Quartile 1 | 2/11 | 1.00 | 1.00 |  |
| Quartile 2 | 5/15 | 2.12 (0.30, 15.15) | 1.10 (0.13, 9.16) |  |
| Quartile 3 | 9/19 | 4.12 (0.66, 25.71) | 2.85 (0.40, 20.52) |  |
| Quartile 4 | 22/33 | 9.38 (1.54, 57.08) | 5.67 (0.74, 43.71) |  |

CI, confidence interval; GDM, gestational diabetes mellitus; OR, odds ratio; SD, standard deviation.

^a^ Adjusted for maternal age (continuous, year), gestational age (continuous, week), and education level (≤12 years and >12 years).

^b^ Adjusted for smoking status (never, former, and current), alcohol consumption (never, former, and current), physical activity (continuous, MET-h∙week-1), baseline BMI (continuous, kg/m^2^), parental history of diabetes (yes and no), history of gestational diabetes (yes and no), parity (0 and ≥1), and variables adjusted for in Model 1.

^c^ *P* for interaction was calculated via the likelihood ratio test by adding an interaction term of a stratifying variable and C-peptide.

**Table S4 Summary statistics to assess the early-pregnancy C-peptide in predicting gestational diabetes mellitus**

|  | C-statistic | | |  | Category-free NRI (%) | |  | IDI | | |
| --- | --- | --- | --- | --- | --- | --- | --- | --- | --- | --- |
|  | Estimates  (95% CI) | Difference | *P* value |  | Value (95% CI) | *P* value |  | Absolute IDI  (95% CI) | *P* value | |
| Conventional predictive factors | 0.63 (0.59, 0.67) | Reference | - |  | Reference | - |  | Reference | | - |
| Conventional predictive factors + C-peptide | 0.66 (0.62, 0.69) | 0.03 | 0.008 |  | 19.6 (6.6, 32.5) | 0.036 |  | 0.018 (0.009, 0.027) | | <0.001 |
| Conventional predictive factors + FBG | 0.65 (0.61, 0.68) | 0.02 | 0.240 |  | 16.0 (2.8, 29.1) | 0.018 |  | 0.023 (0.013, 0.033) | | <0.001 |
| Conventional predictive factors + FBG + C-peptide | 0.66 (0.62, 0.70) | 0.03 | 0.028 |  | 25.9 (12.8, 39.0) | <0.001 |  | 0.033 (0.021, 0.045) | | <0.001 |
| Conventional predictive factors + FBG | 0.65 (0.61, 0.68) | Reference | - |  | Reference | - |  | Reference | | - |
| Conventional predictive factors + C-peptide ^a^ | 0.66 (0.62, 0.69) | 0.01 | 0.412 |  | -6.6 (-19.8, 6.5) | 0.324 |  | -0.005 (-0.017, 0.006) | | 0.377 |
| Conventional predictive factors + FBG + C-peptide ^a^ | 0.66 (0.62, 0.70) | 0.01 | 0.021 |  | 19.9 (6.9, 32.9) | 0.003 |  | 0.010 (0.003, 0.017) | | 0.005 |

CI, confidence interval; FBG, fasting blood glucose; IDI, integrated discrimination improvement; NRI, net reclassification improvement.

Conventional predictive factors included maternal age, gestational age, pre-pregnancy body mass index, physical activity, parental history of diabetes mellitus, and history of gestational diabetes mellitus.

^a^ Compared to conventional predictive factors + FBG.

6,143 pregnant women were recruited into the TSBC in early pregnancy, and underwent 75g 2-h OGTT at 24-28 weeks of pregnancy

347 GDM women were diagnosed according to the IADPSG criteria.

15 participants were excluded:

14 GDM cases did not provide blood sample at enrollment;

1 GDM case had data missing for key covariates.

332 GDM women were included.

664 pregnant women with normal glucose tolerance were selected for individually matching at 1:2 based on maternal age and gestational age.

Final sample size: 996

332 GDM cases

664 matched controls

**Figure S1 Flowchart of participants selection in the nested case-control study.** GDM, gestational diabetes mellitus; IADPSG, International Association of Diabetes in Pregnancy Study Groups; OGTT, oral glucose tolerance test; TSBC, Tongji-Shuangliu Birth Cohort.


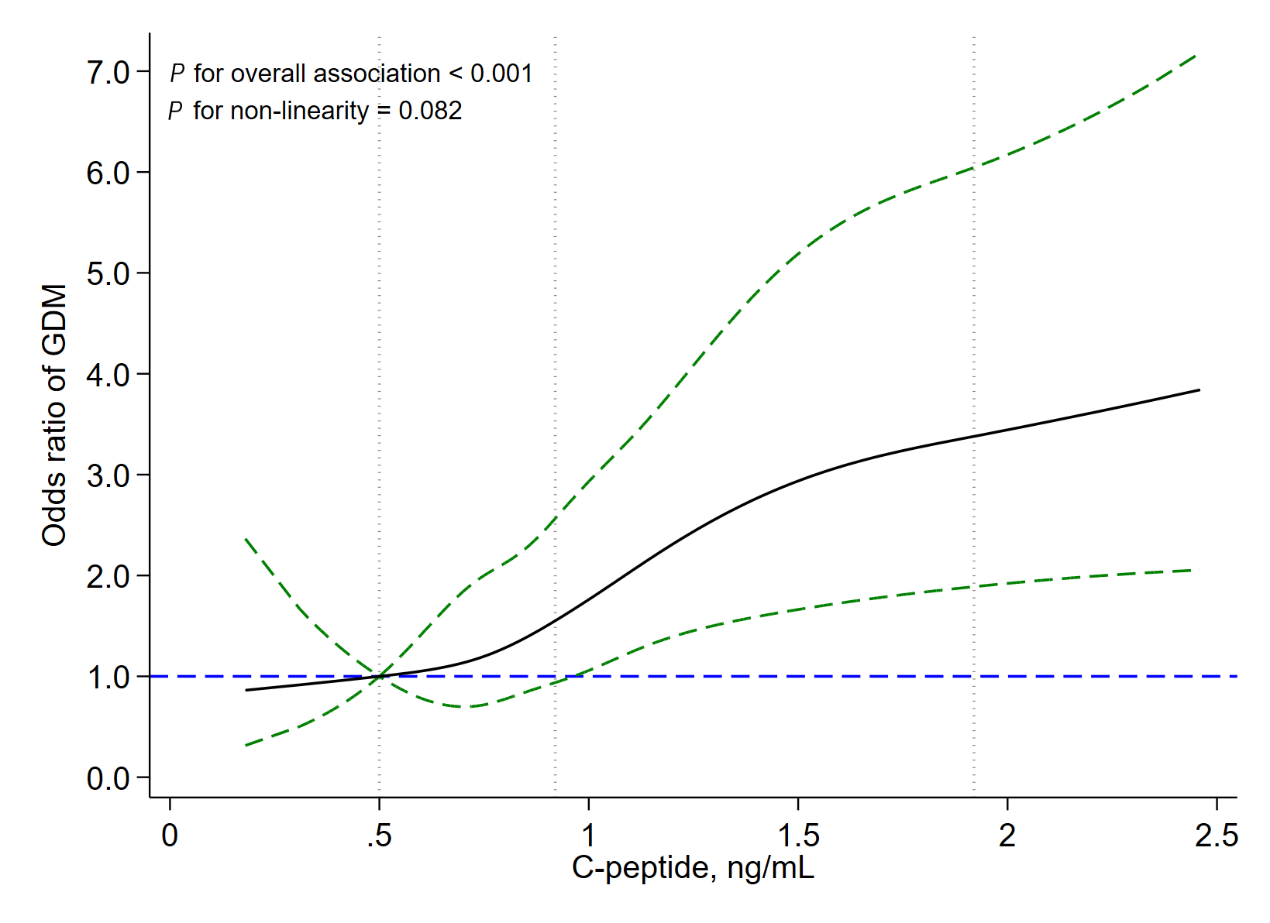


**Figure S2 Restricted cubic splines-based modeling for the association between early-pregnancy serum C-peptide and GDM.** Early-pregnancy C-peptide was positively associated with risk of GDM (*P* for overall association < 0.001), with little evidence for nonlinearity (*P* for nonlinearity = 0.082)**.** The solid line represents estimates of ORs and dashed lines represent 95% CIs, relative to the reference level (5th centile). The dotted vertical lines correspond to the 5th, 50th, and 95th centiles of C-peptide. The ORs were estimated using conditional logistic regression based on restricted cubic splines with adjustment for maternal age (continuous, year), gestational age (continuous, week), education level (≤12 years and >12 years), smoking status (never, former, and current), alcohol consumption (never, former, and current), physical activity (continuous, MET-h∙week-1), pre-pregnancy BMI (continuous, kg/m^2^), parental history of diabetes (yes and no), history of gestational diabetes mellitus (yes and no), and parity (0 and ≥1). CI, confidence interval; GDM, gestational diabetes mellitus; OR, odds ratio.
